# Gender difference in quality of life (QoL) among outpatients with schizophrenia in a tertiary care setting

**DOI:** 10.1186/s12888-021-03051-2

**Published:** 2021-01-28

**Authors:** Saleha Shafie, Ellaisha Samari, Anitha Jeyagurunathan, Edimansyah Abdin, Sherilyn Chang, Siow Ann Chong, Mythily Subramaniam

**Affiliations:** Research Division, Institute of Mental Health, 10 Buangkok View, Singapore, 539747 Singapore

**Keywords:** Schizophrenia, Quality of life, Gender differences

## Abstract

**Background:**

Patients with mental illness report lower quality of life (QoL) compared to the general population. Prior research has found several differences in clinical features and experiences of male and female patients with schizophrenia. Given these differences, it is also important to explore if there are any gender differences in terms of their QoL. This study aimed to investigate differences in QoL between and within each gender among outpatients with schizophrenia in Singapore.

**Methods:**

A total of 140 outpatients were recruited through convenience sampling at the Institute of Mental Health, Singapore. QoL was measured using the brief version of World Health Organization Quality of Life (WHOQOL-BREF) which consists of four domains: physical health, psychological health, social relationships, and environment. QoL scores of males and females were compared using independent t-tests, and multiple linear regressions were used to examine sociodemographic correlates of QoL in the overall sample and within each gender.

**Results:**

There was no significant difference in QoL domain scores between genders. Among males, Indian ethnicity (versus Chinese ethnicity) was positively associated with physical health (β=3.03, *p*=0.018) while males having Technical Education/ Diploma/ A level education (versus Degree and above) were positively associated with social relationships domain (β=2.46, *p*=0.047).

Among females, Malay ethnicity (versus Chinese ethnicity) was positively associated with physical health (β=1.95, *p*=0.026) psychological health (β=3.21, *p*=0.001) social relationships (β=2.17, *p*=0.048) and environment (β=2.69, *p*=0.006) domains, while females who were separated/divorced (versus single) were inversely associated with psychological health (β=− 2.80, *p*=0.044) and social relationships domains (β=− 4.33, *p*=0.011). Females who had Secondary and below education (versus Degree and above) were inversely associated with social relationships (β=− 2.29, *p*=0.028) and environment domains (β=− 1.79, *p*=0.048).

**Conclusions:**

The findings show the importance of treatments targeting QoL to attend to both the clinical features of the illness as well patient’s sociodemographic characteristics.

## Background

Schizophrenia is a debilitating mental illness and is one of the leading contributors to the global burden of disease [1, 2]. Individuals with schizophrenia face significant clinical and psychosocial challenges and have been reported to experience poorer quality of life (QoL) as compared to the general population [3–6].

QoL can be broadly defined as a multi-dimensional concept that describes an individual’s perceptions of physical, psychological, and social well-being, as well as his or her relationship to important features of their environment [7]. QoL is used both as a measure of functioning and recovery in patients with mental disorders and to evaluate the treatment provided [8]. Researchers have argued that aside from focusing on treating symptoms of mental illnesses, care and treatment provided to patients should also address factors that can improve QoL. Notably, the level of unmet needs has been found to be the best predictor of QoL [9].

Prior studies which have examined QoL among patients with schizophrenia in Singapore include studies measuring the neurocognitive, clinical, and functional correlates of QoL (measured using World Health Organisation Quality of Life Assessment-Brief Form) [10], relationship of psychosocial factors with QoL (measured using 12-Item Short Form Health Status Questionnaire) [11], relationship of schizophrenia patients’ self-esteem and QoL with perceived stigma and coping orientations (measured using Quality of Life Questionnaire in Schizophrenia (S-QoL)) [12], and a community study comparing QoL of schizophrenia outpatients with general practice outpatients (measured using Dartmouth COOP – World Organization of Family Doctors Functional Health Assessment Charts (COOP-WONCA charts)) [3].

While schizophrenia affects both males and females alike, prior research has noted several differences in its clinical features and experiences between males and females with schizophrenia. They found that males have an earlier age of onset [13–16], poorer premorbid functioning, worse prognosis [16], and exhibit more negative symptoms [13, 16]. On the other hand, females were found to have better course of illness in the short and middle term, display more affective symptoms, auditory hallucinations and persecutory delusions [16], and have a better treatment response to typical antipsychotics [17] and antipsychotics in the pre- menopausal period, though this comes with increased side effects [16].

Given these differences between males and females with schizophrenia, it is also important to explore if there are any gender differences in terms of their QoL. Mixed results were seen in the literature where some found no gender difference [18, 19] while others found that either males [20, 21] or females had better QoL [22, 23]. Examples of studies which reported males having better QoL include studies done by Kujur et al. [20] and Chan and Yu [21]. Kujur et al. [20] used the Hindi version of WHOQOL-BREF to measure QoL among 60 patients diagnosed with schizophrenia in a hospital setting in India, while Chan and Yu [21] used the Chinese version of WHOQOL-BREF to measure QoL among 172 patients diagnosed with schizophrenia for at least two years from a psychiatric outpatient department in a hospital in Hong Kong. Studies which found that females had better QoL include studies done by Silva et al. [22] and Salokangas et al. [23]. Silva et al. [22] explored QoL among a sample of 123 deinstitutionalized psychiatric patients with schizophrenia from a Mental Health Reference Service (SERSAM). The authors used the QLS-BR scale which was developed in Brazil to measure QoL among patients with schizophrenia. Salokangas et al. [23] on the other hand explored QoL among a sample of 3256 long-term schizophrenia patients discharged from mental hospitals in Finland. These patients were interviewed using a structured interview specifically designed for the study upon three years of being discharged. It is important to note that differences in findings across studies may be partly explained by the complex construct of QoL [24], variations in study design, sample size, characteristics of the population, and tools used to measure QoL.

While researchers have generally explored various socio-demographic (e.g. gender, age, marital status, education) and clinical (e.g. negative symptoms, positive symptoms, presence of other mental illness) determinants of QoL among patients with schizophrenia [24–26], less attention has been paid towards the examination of these determinants within each gender [27]. Comparisons within each gender are important to better understand its epidemiology and therefore provide more tailored and suitable interventions to improve their QoL. When comparisons are made only between gender, differences that may exist within each gender are overlooked.

Therefore, to address these limitations, this study sought to investigate differences in QoL between and within each gender among a multi-ethnic population of outpatients with schizophrenia in Singapore.

## Methods

Data collected was part of a cross-sectional survey, which was described in an earlier paper [28] and conducted from October 2015 to December 2016 at the Institute of Mental Health (IMH) to explore the pathways to care amongst outpatients with mental illnesses. IMH is the only tertiary care hospital in Singapore serving those with mental illnesses. Patients recruited in this study were seeking treatment at IMH outpatient clinics and their affiliated satellite clinics. As patients were recruited from an outpatient group, they were patients with generally milder symptoms of schizophrenia as compared to those seeking inpatient care. Ethics approval for the study was given by the National Healthcare Group Domain Specific Review Board. Written consent was obtained from all participants prior to taking part in the study.

One hundred forty outpatients were recruited through convenience sampling at IMH. Inclusion criteria were Singapore residents (including Singapore Citizens and Permanent Residents); those aged 21–65 years old; belonging to Chinese, Malay or Indian ethnic groups; able to provide consent; able to understand and read English, Chinese, Malay or Tamil; and have a clinical diagnosis of schizophrenia and related psychoses with a duration of illness of not more than 2 years as determined by a psychiatrist using International Classification of Diseases 9th Revision (ICD-9R) criteria. Participants excluded were those who had intellectual disabilities or cognitive impairment, or those who had been seeking treatment for mental health problems for more than 2 years, or those who are not fluent in English, Chinese, Malay or Tamil language.

### Main instruments

#### WHOQOL-BREF

QoL was measured using the brief version of World Health Organization Quality of Life (WHOQOL-BREF) which consists of four domains: physical health, psychological health, social relationships, and environment [29]. It is a 26- item self-administered questionnaire which is used to measure an individual’s perception of quality of life over the past two weeks. Two questions, apart from the four domains, assess overall perception of QoL (OPQOL) and overall perception of health (OPH). All responses were measured on several variations of a 5-point Likert scale, with scores from 1 to 5, enquiring on “how much”, “how completely”, “how good or satisfied” or “how often” the individual felt or experienced certain things over the past two weeks. Mean scores are derived for each domain. This scale has been validated for use in the Singapore population [30].

#### Socio-demographic questionnaire

This questionnaire collected information on the participant’s age, gender, ethnicity, marital status, educational status, and employment status.

### Statistical analysis

All statistical analyses were performed using Statistical Package for Social Sciences (SPSS) version 23. Descriptive statistics were computed for the basic demographic and clinical variables. Mean and standard deviations (SD) were calculated for continuous variables, and frequencies and percentages for categorical variables. Chi-square tests and t-tests were conducted to explore any significant differences in the categorical (ethnicity, marital status, education and employment status) and continuous (age) variables respectively between males and females.

QoL scores of males and females were compared using independent t-tests, and multiple linear regressions were used to examine sociodemographic correlates of QoL in overall sample and within each gender. Two-sided statistical significance was set at *p* value < 0.05.

## Results

A total of 140 participants with schizophrenia and related psychoses were recruited. The mean age of participants was 31.7 (SD = 9.3) years, ranging from 21 to 63 years, with almost equal number of males (51.4%) and females (48.6%). The majority of participants were Chinese (63.6%), single (70.7%), had Technical Education/ Diploma/ A level education (45.7%), and were unemployed (57.1%). The socio-demographic characteristics of the sample can be found in Table 1.

**Table 1:** Socio-demographic details of participants (*N* = 140)

		Overall		Male		Female		
		N	%	N	%	N	%	*p* value*
Age	min=21; max=63 mean=31.7; SD=9.3	140		72		68		0.999
Ethnicity	Chinese	89	63.6	46	63.9	43	63.2	0.248
	Malay	34	24.3	20	27.8	14	20.6	
	Indian	17	12.1	6	8.3	11	16.2	
Marital status	Single	99	70.7	55	76.4	44	64.7	0.189
	Married	30	21.4	11	15.3	19	27.9	
	Separated/Divorced	11	7.9	6	8.3	5	7.4	
Education	Secondary and below	46	32.9	22	30.6	24	35.3	0.026
	Technical Education/ Diploma/ A level	64	45.7	40	55.6	24	35.3	
	Degree and above	30	21.4	10	13.9	20	29.4	
Employment status	Employed	60	42.9	35	48.6	25	36.8	0.157
	Unemployed	80	57.1	37	51.4	43	63.2	

*Chi-square test for comparison between males and females for categorical variables (ethnicity, marital status, education and employment status); t-test for comparison between males and females for continuous variable (age)

The t-tests showed that there was no significant difference in OPQOL, OPH and QoL domain scores between genders (Table 2). Multiple linear regression analysis conducted found no gender differences across all 4 WHOQOL-BREF domains after controlling for all other sociodemographic factors (Table 3).

**Table 2:** Scores on WHOQOL-BREF

		Overall			Male			Female		
	N	mean	SD	N	mean	SD	N	mean	SD	*p* value*
Overall perception of QOL (OPQOL)	140	3.39	0.9	72	3.31	0.9	68	3.49	0.9	.257
Overall perception of health (OPH)	140	3.24	1.0	72	3.29	1.0	68	3.19	1.1	.564
**QOL domains:**										
Physical health	140	13.61	2.7	72	13.34	2.8	68	13.89	2.6	.235
Psychological health	140	12.89	3.1	72	12.67	3.1	68	13.13	3.1	.377
Social relationships	138	12.57	3.4	71	12.23	3.3	67	12.94	3.5	.228
Environment	140	13.64	2.9	72	13.43	2.9	68	13.86	3.0	.389

*t-test between males and females

**Table 3:** Socio-demographic correlates of the WHOQOL-BREF domains for the overall sample

	Physical health				Psychological health				Social relationships				Environment			
	B	*p* value	95% C.I.		B	*p* value	95% C.I.		B	*p* value	95% C.I.		B	*p* value	95% C.I.	
Gender*																
Male	−.687	.152	−1.630	.256	−.815	.117	−1.837	.207	−1.060	.071	−2.213	.093	−.616	.230	−1.627	.394
Female	Ref				Ref				Ref				Ref			

*Multiple linear regression conducted between gender and QoL domains (physical health, psychological health, social relationships, and environment) while controlling for ethnicity, marital status, employment status, education, and age

Statistically significant *p* values (*p*< 0.05)

Table 4 (among males) and Table 5 (among females) show the multiple linear regression analyses on the correlates of the WHOQOL-BREF domains stratified by gender which indicate several sociodemographic correlates to be significant. Among males, Indian ethnicity (versus Chinese ethnicity) was positively associated with physical health (β=3.03, *p*=0.018) while males having Technical Education/ Diploma/ A level education (versus Degree and above) were positively associated with social relationships domain (β=2.46, *p*=0.047).

**Table 4:** Socio-demographic correlates of the WHOQOL-BREF domains among males

	Physical health				Psychological health				Social relationships				Environment			
	B	*p* value	95% C.I.		B	*p* value	95% C.I.		B	*p* value	95% C.I.		B	*p* value	95% C.I.	
**Ethnicity**																
Malay	0.614	0.428	−0.923	2.151	0.991	0.259	−.0747	2.730	−0.141	0.878	−1.969	1.687	0.132	0.871	−1.480	1.743
Indian	3.028	**0.018**	0.535	5.521	2.705	0.060	−0.114	5.524	0.368	0.805	−2.601	3.338	1.057	0.422	−1.556	3.671
Chinese	Ref				Ref				Ref				Ref			
**Marital status**																
Married	0.572	0.622	−1.733	2.878	0.527	0.688	−2.080	3.134	0.083	0.952	−2.661	2.827	0.208	.864	−2.208	2.625
Separated/ Divorced	−1.325	0.380	−4.320	1.669	−1.927	0.260	−5.313	1.459	−2.290	0.203	−5.846	1.267	−1.401	.376	−4.540	1.738
Single	Ref				Ref				Ref				Ref			
**Employment status**																
Employed	0.620	0.369	−0.748	1.988	0.791	0.310	−0.755	2.338	−0.353	0.668	−1.988	1.282	0.166	.818	−1.268	1.600
Unemployed	Ref				Ref				Ref				Ref			
**Education**																
Secondary and below	−0.566	0.622	−2.850	1.718	0.047	0.971	−2.536	2.630	0.419	0.759	−2.293	3.131	−1.361	.260	−3.755	1.034
Technical Education/ Diploma/ A Levels	1.000	0.331	−1.039	3.038	1.300	0.264	−1.004	3.605	2.462	**0.047**	0.035	4.888	0.838	.436	−1.299	2.975
Degree and above	Ref				Ref				Ref				Ref			
**Age**	0.001	0.978	−0.103	0.105	0.013	0.828	−0.105	0.130	0.001	0.989	−0.123	.125	0.010	.852	−.099	.119

Bold - Statistically significant p values (*p*< 0.05)

**Table 5:** Socio-demographic correlates of the WHOQOL-BREF domains among females

	Physical health				Psychological health				Social relationships				Environment			
	B	*p* value	95% C.I.		B	*p* value	95% C.I.		B	*p* value	95% C.I.		B	*p* value	95% C.I.	
**Ethnicity**																
Malay	1.947	.**026**	.242	3.653	3.211	**.001**	1.429	4.993	2.166	.**048**	.016	4.315	2.693	**.006**	.819	4.567
Indian	−.391	.671	−2.227	1.445	.427	.658	−1.491	2.344	−.679	.571	−3.065	1.706	−.282	.781	−2.299	1.735
Chinese	Ref				Ref				Ref				Ref			
**Marital status**																
Married	.389	.657	−1.355	2.133	.708	.440	−1.115	2.530	.058	.958	−2.140	2.257	−.150	.876	−2.067	1.766
Separated/ Divorced	−1.951	.140	−4.563	.661	−2.804	**.044**	−5.533	−.075	−4.329	**.011**	−7.625	−1.032	−2.671	.068	−5.542	.199
Single	Ref				Ref				Ref				Ref			
**Employment status**																
Employed	.521	.449	−.845	1.887	1.108	.126	−.320	2.535	.796	.361	−.935	2.526	.654	.387	−.847	2.156
Unemployed	Ref				Ref				Ref				Ref			
**Education**																
Secondary and below	−1.290	.115	−2.905	.324	−1.100	.197	−2.786	.587	−2.294	.**028**	−4.329	−.260	−1.787	**.048**	−3.562	−.013
Technical Education/ Diploma/ A Levels	.093	.909	−1.532	1.717	1.239	.149	−.458	2.936	.261	.800	−1.797	2.320	−.787	.381	−2.572	.998
Degree and above	Ref				Ref				Ref				Ref			
**Age**	−.013	.770	−.101	.075	.071	.127	−.021	.163	.055	.327	−.056	.166	−.020	.680	−.117	.077

Bold - Statistically significant p values (*p*< 0.05)

Among females, Malay ethnicity (versus Chinese ethnicity), was positively associated with physical health (β=1.95, *p*=0.026), psychological health (β=3.21, *p*=0.001), social relationships (β=2.17, *p*=0.048), and environment (β=2.69, *p*=0.006) domains, while females who were separated/divorced (versus single), were inversely associated with psychological health (β=− 2.80, *p*=0.044) and social relationships domains (β=− 4.33, *p*=0.011). Females who had Secondary and below education (versus Degree and above) were inversely associated with social relationships (β=− 2.29, *p*=0.028) and environment domains (β=− 1.79, *p*=0.048).

## Discussion

The present study sought to examine differences in QoL between and within each gender among a sample of outpatients with schizophrenia in Singapore using the WHOQOL-BREF. In general, significant differences across the WHOQOL-BREF domains were seen within each gender but not between genders.

There were several studies which have examined gender difference in QoL among the general population in Singapore. These include studies done by Abdin et al. [31] who used the EuroQol- 5 Dimension (EQ-5D), as well as Thumboo et al. [32] and Leow et al. [27] who used the 36-Item Short Form Health Survey questionnaire (SF-36). Only Leow et al. [27] found significant difference in QoL scores between genders whereby females scored lower than males in the physical domain of QoL.

However, no significant differences between males and females were found across any of the WHOQOL-BREF domains (physical health, psychological health, social relationships and environment) in this study. This replicates the findings from a study in Taiwan among 148 individuals with schizophrenia living in the community using WHOQOL-BREF and its Chinese version [6]. On the contrary, there are studies which found significant gender difference in at least one of the four domains of WHOQOL-BREF. For example, a study conducted in Hong Kong and China [33] found that males reported better subjective QoL in the physical health domain compared to females but found no significant gender difference in the other three domains of QoL. The authors conjectured that distressing factors such as severe discrimination in Chinese society towards females with schizophrenia may have negatively influenced their QoL.

Results from our study found significant differences in QoL based on socio-demographic variables within each gender. However, considering that few studies have examined differences in QoL within each gender among both the general population and patients with schizophrenia, discussion and comparison of the present findings are limited.

Females in the present sample who were separated or divorced scored significantly lower in the psychological health and social relationships domains of QoL compared to females who were single. However, while males who were separated or divorced had similarly lower scores in these domains than those who were single, these differences were not statistically significant. Nonetheless, this pattern was also observed in a sample of patients with schizophrenia attending community mental health services in Poland where those who were divorced and widowed reported significantly lower scores in the psychological health, social relationships, and environment domains compared to those who were unmarried or married [34]. In general, evidence from previous studies suggests that those who were divorced (both males and females) experience significant challenges resulting from the dissolution of their marriages. These include adverse health consequences [35], feelings of loss [36], and having to face a lot of different social-relational and socio-economic changes after divorce [37]. The challenges that accompany dissolution of marriages can be culture specific. For example, a qualitative study conducted specifically among a sample of female patients with schizophrenia in India whose marriages had dissolved reported facing significant levels of stigma attached to the separation. This is due to marriage being revered in the Indian society and is associated with high levels of social approval [38].

In an Asian community where a strong emphasis is placed on interdependence of family members and the maintenance of harmonious familial relationship, having been divorced or separated may affect the perception of social relationships strongly. We thus speculate that the effect of marital status on QoL within females is also perhaps more pronounced and significant compared to males as females are expected to be dependent on family as compared to males [39].

Malay females reported significantly better QoL as compared to Chinese females across all four WHOQOL-BREF domains. However, differences in QoL between Chinese and Malay males were not significant. Similarly, while Indian males reported significantly better QoL than Chinese males in the physical health domain, this effect was not seen between Indian and Chinese females. Studies examining the effect of ethnicity on QoL in Singapore were largely conducted among the general population. An earlier study which had explored the effect of ethnicity on QoL among patients with schizophrenia found that the Chinese reported better physical functioning scores as compared to the Malays and Indians [40]. No examination within each gender was conducted. On the other hand, a study by Leow et al. [27] who did examine the effect of QoL within each gender among the general population found contrasting results whereby Chinese females had reported better scores in the physical health domain of QoL compared to the Malay and Indian females.

Different cultural expectations, lifestyle practices, and social circumstances for females and males within each ethnicity [41] could be a possible explanation for why the effect of ethnicity on QoL is different in females and males. For example, Jung et al. [41] posited that variation in gender roles in the Malay culture is still fairly prominent. The authors reason that Malay females still generally subscribe to the conservative view of being submissive in the household and take on the full-time role of taking care of their children while Chinese and Indian females are gradually shifting away from these conservative views on masculinity and traditional gender roles. Similarly, Abdullah et al. [42] also mentioned that while many Malay females are presently employed, they are still expected to remain oriented towards family matters and adhere to traditional views of a woman. Prior studies have noted variations in the way different ethnic groups perceive and respond to mental illness. Based on their research findings on stigma towards mental illness, Subramaniam et al. [43] suggested that the Malays may be more tolerant and accepting of people with mental illness. They posited that this tolerance could be due to the tenets of Islam, a religion the majority of Malays in Singapore identify with, which perceives mental illness as a test from God [44, 45]. In addition, Sands [46] mentioned that Malay families were more likely to accept the patient’s illness and were more willing to relieve them of their obligations towards family and social roles. In line with this argument, it is possible that Malay females with schizophrenia in this sample who initially were more submissive in the household and had greater obligations towards family matters as compared to Malay males, were no longer expected to maintain them to the same extent as before they had their illness or were receiving support in carrying out their roles. This shift in responsibility may have alleviated the burden of the illness significantly and consequently their QoL. However traditional views and social roles may have changed over time and the extent to which individuals in the present sample identify with their respective cultural values and practices were not gathered in this study. In addition, social support received was also not measured in this study. These collectively render any conclusion limited and speculative at best. Future studies are therefore needed to examine the extent to which individuals from different ethnic backgrounds identify with their respective cultural values and practices, the social support received, as well as differences in lifestyle practices and social circumstances in relation to gender roles to understand this variance in QoL better.

Employment plays a central role in providing financial income and non-financial benefits yet unemployment is one of the biggest issues facing patients with schizophrenia. Importantly, employment has been found to be a strong predictor of QoL in several studies whereby those who are employed have higher QoL than those who are unemployed [47–50]. In addition, a study by Üçok et al. [47] who examined the relationship between work/study status, symptom severity, functionality, and quality of life in schizophrenia patients found that those who work/study full-time had less severe negative symptoms, better functioning, and higher levels of QoL. Understandably, severity of negative symptoms can affect many aspects of daily functioning, including an individual’s functional abilities to work, and subsequently their QoL. Similarly, another study conducted by Guedes de Pinho et al. [48] amongst patients who were diagnosed with schizophrenia and receiving treatment at the psychiatry services of eight hospitals in Portugal found that being employed was associated with higher quality of life in three out of the four domains of the WHOQOL BREF (physical health, psychological health, and environment).

However, unlike these studies, no significant differences in QoL were seen between those who were employed and those who were unemployed in both male and female populations in the current study. A probable explanation for these findings is that those who were unemployed in this sample may be receiving sufficient welfare support that allows them to manage their day to day living. Furthermore, most patients with schizophrenia in an Asian setting live with their families thereby suggesting the possibility of receiving sufficient familial support to substitute the benefits of employment. However, such conclusions may be stronger with the presence of data on social or welfare support that patients are receiving, which were not collected in the present study. Future studies should explore the relationship between employment and social support on QoL. There is a concern on large number of intra-group statistical tests in a study with low sample size. We found that for multiple linear regression analysis, the “two subjects per variable” rule of thumb is commonly used to estimate sample size required [51]. Based on this rule of thumb, for this study, the minimum sample size of 16 would be required for a linear regression with 8 predictors included. Therefore, we consider our sample size acceptable for using statistical tests within each group (male = 72, female =68). However, we are unable to deny that this approach might not reflect a real-world complexity of the relationship between variables.

The present study has several limitations. Firstly, patients in the sample were outpatients and comprise those who had started receiving treatment within the last two years, therefore limiting the generalizability to all patients with schizophrenia. Comparisons across other samples should thus be made with caution. Furthermore, the present study does not examine any clinical variables and therefore any interaction analysis with clinical factors was not possible. This may limit the depth of the present findings since clinical factors have been consistently found to be associated with functioning and QoL among patients with schizophrenia in other studies. Future research should examine the interaction effects between gender and clinical variables on outcomes. Thirdly, the number of Malay and Indian participants in the present sample is small and may have led to a biased result. Readers are thus advised to interpret the results with caution. Future research examining ethnic differences in this domain should aim to recruit a more proportionate sample for each ethnicity. Lastly, while the WHOQOL-BREF allows comparison of QoL with various populations, it is a generic instrument which may not be sensitive enough to detect changes of QoL among patients with schizophrenia.

Nonetheless, the present research reduces the gap in the literature in terms of knowledge on differences within each gender and points to the importance of understanding these gender-specific nuances in patients’ socio-demographic background on patient outcomes. Specifically, it highlights the differences that exist within a specific gender and that different socio-demographic variables may contribute to different outcomes on males and females. Findings from the study also suggests that delivery of services or interventions targeted at female patients should pay attention to those who are separated/divorced, or those with lower education, given that they have poorer quality of life in the various domains of the WHOQOL-BREF.

## Conclusions

In an ideal world, each patient is given a treatment that is unique to them because no two patients are identical but practicing that in the present situation may be difficult. Differentiating within men and women is another step to creating a more holistic treatment approach that is sensitive to each individual’s background. Thus, aside from addressing clinical features and reducing symptoms of the illness, an ideal treatment would also focus on understanding how a patient’s socio-demographic background may affect adjustment and coping of the illness on day to day living activities, and subsequently their QoL. Knowledge on how clinical factors interact with different socio-demographic variables within males and females would also be useful for clinical services and researchers aiming to develop gender-specific interventions.

## Data Availability

The datasets used and/or analysed during the current study are available from A/Prof Mythily Subramaniam (mythily@imh.com.sg) on reasonable request.

## Abbreviations

COOP-WONCA charts: COOP – World Organization of Family Doctors Functional Health Assessment Charts
EQ-5D: EuroQol- 5 Dimension
ICD-9R: International Classification of Diseases 9th Revision
IMH: Institute of Mental Health
OPH: overall perception of health
OPQOL: overall perception of QoL
QoL: Quality of Life
S-QoL: Quality of Life Questionnaire in Schizophrenia
SD: standard deviations
SF-36: 36-Item Short Form Health Survey questionnaire
SPSS: Statistical Package for Social Sciences
WHOQOL-BREF: Brief version of World Health Organization Quality of Life
